# Supplementary material for: Cognitive expertise in esport experts: a three-level model meta-analysis
Source: PeerJ. 2024 Aug 7;12:e17857. doi: 10.7717/peerj.17857 (PMC11316462; doi:10.7717/peerj.17857)
Supplement: Supplemental Information 2 [file peerj-12-17857-s002.docx]

**Table S1 Definition of Expert or amateur group.**

| **Professional level** | **Expert group definition** | **Amateur group definition** |
| --- | --- | --- |
| professionals vs. Amateurs | Experts were either registered professional players, part of an organized team (Regardless of their ranking). | Non-professional players, ranked below the top 1%. |
| high-ranked vs.Amateurs | high-ranked non-professional players (top 1%). | Non-professional players, ranked below the top 1%. |

**Table S2 Inclusion and exclusion criteria of meta-analysis.**

|  | **Inclusion criteria** | **Exclusion criteria** |
| --- | --- | --- |
| 1.Player Qualifications: experts | Experts were either registered professional players, part of an organized team, or high-ranked non-professional players. :top 1%. | Experts were only defined without ranking information or professional qualifications:e.g. self-created questions or game experience. |
| 2.Player Qualifications: amateurs | Amateurs had never played in tournaments and possessed lower rankings than experts. | Novice with no game experience. |
| 3.Game genre | Any competitive video game with open tournaments, including FPS, TPS, RTS, Fight games, Sports games, MOBA, Mobile, or other game genre:e.g. Hearthstone | Casual games or gams have no open tournaments |
| 4.Cognitive indicators | Employed at least one paradiam to assess cognitive abilities, also including simple reaction time or motor control. | Only investigated vision or gaze behavior. |
| 5.Research topics | Compared the experts’ cognitive abilities with amateurs. | Compared experts solely with novices. Game intervention studies. |
| 6.Other | Full-text articles with versions available English or other languages. | Statistics are incomplete. No response from emails. |

**Table S3 Esports games classification**

| **Game genre** | **m** | **k** | **Study** | **Games** |
| --- | --- | --- | --- | --- |
| Shooting games | 2 | 27 | Benoit et al., 2020 | OW, CS, COD,Rainbow 6, PUBG, Battlefield 4 |
|  |  |  | Cretenoud et al., 2021 | CSGO |
| Strategy games | 6 | 58 | Ding et al., 2018 | LOL |
|  |  |  | Gao, 2018 | LOL |
|  |  |  | Kim et al., 2023 | SC |
|  |  |  | Li et al., 2020 | LOL |
|  |  |  | Röhlcke et al., 2018 | DOTA2 |
|  |  |  | Valls-Serrano et al.,2022 | LOL |
| Mix games | 5 | 47 | Wang et al., 2022 | LOL,PUBG, PUBG: Mobile, Aritifact, Popkart |
|  |  |  | Bickmann et al., 2021 | MOBA, FPS |
|  |  |  | Ferm & Galle, 2013 | LOL; CSGO |
|  |  |  | Kang et al., 2020 | LOL, OW, BG, SC |
|  |  |  | Pluss et al., 2020 | LOL, HOS, OW, PUBG |
| Other games | 2 | 10 | Tanaka et al., 2013 | Guilty Gear |
|  |  |  | Phillips & Green, 2023 | Fighting; Rhythm Games |

Notes: OW: Overwatch, CS :Counter-Strike, CSGO: Counter-Strike: Global Offensive, COD: Call of Duty, PUBG: PlayerUnknown's Battlegrounds, LOL :League of Legends, HOS :Heroes of the Storm, Popkart: Crazyracing Kartrider.

**Table S4 Cognitive Test Categories**

| Cognitive classification | Paradigm |
| --- | --- |
| 1.perception | Freiburg visual acuity, Time perception... |
| 2.bottom-up attention | Simple reaction time, visual search, Sustained attention... |
| 3.top-down attention | Antisaccade task, Attention blind task, Multiple-object tracking... |
| 4.spatial cognition | Visual Working Memory Task, Mental Rotation... |
| 5.task-switching/multitasking | Number-Letter Task, D-KEFS Color-Word Switching... |
| 6.inhibition | Stop Signal Task, Flanker, Go/No-go... |
| 7.problem solving | Tower of London, WAIS-IV Visual Puzzles... |
| 8.verbal cognition | verbal cognition,WAIS-IV Digit Span... |
| 9.motor control | Manual dexterity, Pursuit Rotor... |

**Table S5 Boolean expression**

| Theme | Boolean expression |
| --- | --- |
| Experts participants | experts OR professional players OR top players OR elite players OR gamers |
| Cognitive abilities | cognition OR cognitive OR perception OR perceptual OR attention OR attentional OR working memory OR executive function OR executive control OR inhibition OR task switching OR multitasking OR Multiple-Object Tracking OR processing speed OR reasoning OR planning OR problem solving OR decision making |
| Games | video game OR esports OR computer game OR Mobile game |

**Table S6 Data from getdata**

| **Study** | **Data from getdata** | **Data in research** |
| --- | --- | --- |
| Benoit et al., 2020 | 4 | 84 |
| Bickmann et al., 2021 | 0 | 36 |
| Cretenoud et al., 2021 | 0 | 24 |
| Ding et al., 2018 | 28 | 28 |
| Ferm & Galle, 2013 | 0 | 96 |
| Gao, 2018 | 0 | 12 |
| Jin et al., 2020 | 0 | 16 |
| Kim et al., 2023 | 16 | 40 |
| Li et al., 2020 | 12 | 108 |
| Phillips & Green, 2023 | 0 | 32 |
| Pluss et al., 2020 | 0 | 32 |
| Röhlcke et al., 2018 | 0 | 12 |
| Tanaka et al., 2013 | 8 | 8 |
| Valls-Serrano et al.,2022 | 0 | 32 |
| Wang et al., 2022 | 0 | 8 |
| Total | 68:11.97% | 568 |

**Table S7 Leave one out analysis.**

| ID | Study | Effect size change | *p* |
| --- | --- | --- | --- |
| 1 | Benoit et al., 2020 | 0.017 | .939 |
| 2 | Bickmann et al., 2021 | 0.030 | .889 |
| 3 | Cretenoud et al.,2021 | 0.048 | .819 |
| 4 | Ding et al., 2018 | 0.019 | .932 |
| 5 | Ferm & Galle., 2013 | 0.027 | .901 |
| 6 | Gao, 2018 | 0.054 | .796 |
| 7 | Kang et al., 2020 | 0.006 | .980 |
| 8 | Kim et al., 2022 | 0.007 | .976 |
| 9 | Li et al., 2020 | 0.003 | .989 |
| 10 | Pluss et al., 2020 | 0.008 | .973 |
| 11 | Phillips & Green, 2023 | 0.046 | .829 |
| 12 | Röhlcke et al., 2018 | 0.022 | .921 |
| 13 | Tanaka et al., 2013 | 0.126 | .469 |
| 14 | Wang et al., 2022 | 0.006 | .976 |
| 15 | Valls-Serrano et al., 2022 | 0.010 | .963 |

**Table S8 Risk of bias.**

| Risk  of bias | Selection of  participants | Measurement  of exposure | Blinding of  outcome assessments | Incomplete  outcome data | Selective  outcome reporting | Confounding  variables |
| --- | --- | --- | --- | --- | --- | --- |
| Low | 15:32% | 0:0% | 0:0% | 9:19% | 15:32% | 8:17% |
| Unclear | 0:0% | 15:41% | 11:30% | 6:16% | 0:0% | 5:14% |
| High | 0:0% | 0:0% | 4:67% | 0:0% | 0:0% | 2:33% |

*
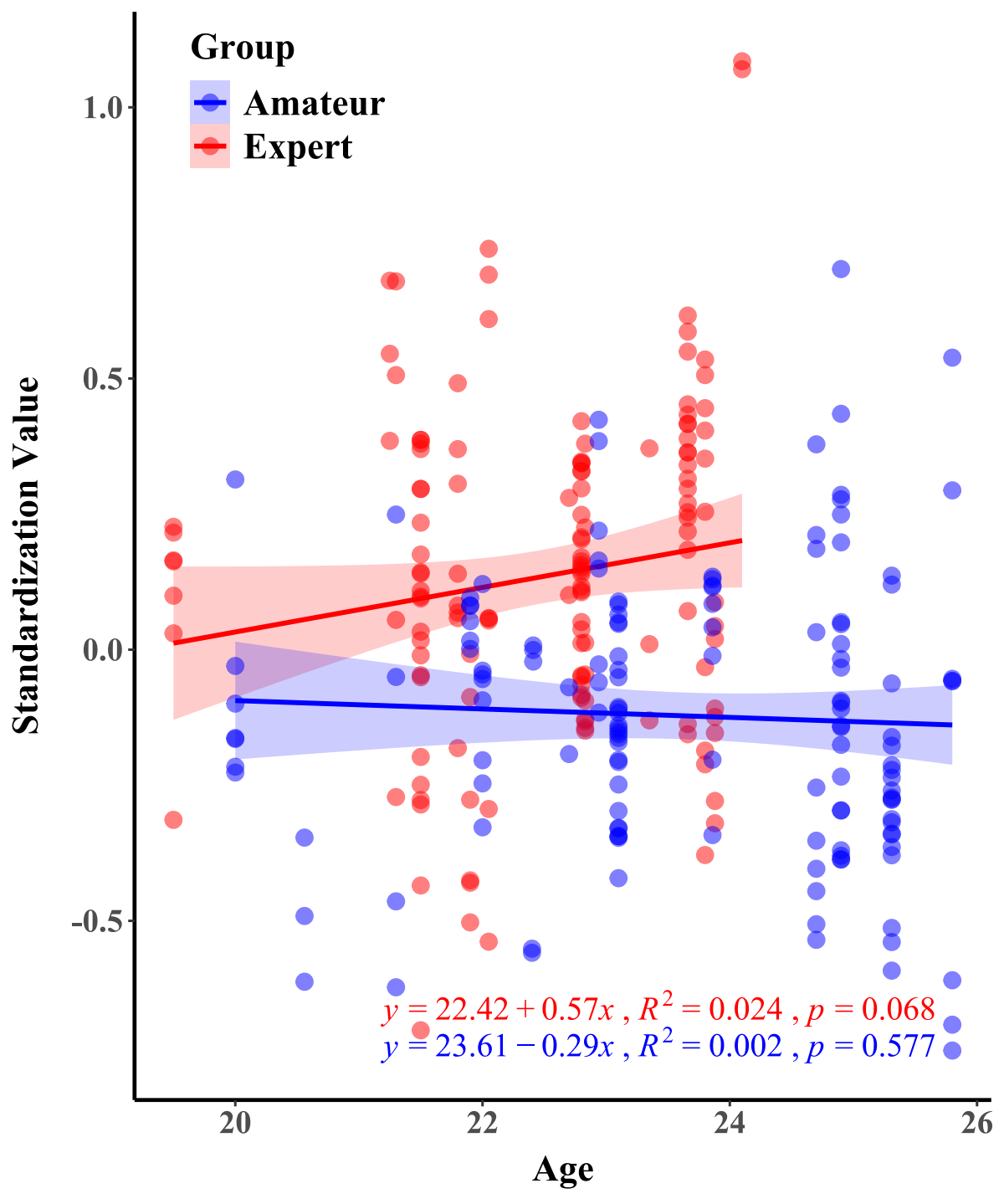
*

**Fig.S1.** Regression analysis of the age of experts and amateurs on cognitive expertise
